# The effect of television advertising on gambling behaviour: a quasi-experimental study during the 2022 Qatar FIFA World Cup

**DOI:** 10.1016/j.abrep.2026.100666

**Published:** 2026-01-13

**Authors:** Ellen McGrane, Robert Pryce, Matt Field, Luke Wilson, Elizabeth Goyder

**Affiliations:** a Sheffield Centre for Health and Related Research (SCHARR), University of Sheffield, Regent Court, 30 Regent Street, Sheffield S1 4DA, United Kingdom; b School of Psychology, University of Sheffield, ICOSS Building, 219 Portobello, Sheffield S1 4DP, United Kingdom

**Keywords:** Gambling, Advertising, Econometrics, Quasi-experiment, Policy

## Abstract

**Introduction:**

Gambling is a recognised public health problem, and population-level advertising restrictions may be an effective way to reduce gambling-related harm. This study fills an important evidence gap by employing a quasi-experiment to estimate the impact of television gambling advertising on gambling behaviour during the 2022 Qatar World Cup. It exploits the variation in gambling advertising between two broadcasters to evaluate its impact on gambling behaviour in a real-world setting.

**Methods:**

Poisson and Logistic panel data regression models using the broadcaster in place of gambling advertising are employed with individual fixed effects and match-level controls. Betting data is from a purposive sample of 365 men aged 18 to 45 who bet on football in England.

**Results:**

Frequency of betting on football was 16 % to 24 % higher during games televised on a channel with gambling advertising compared to one without [IRR: 1.16 – 1.24,p < 0.01]. Furthermore, participants were 22 % to 33 % more likely to place a football bet during games that contained television gambling advertising [OR: 1.22 – 1.33, p < 0.01].

**Conclusions:**

Using a pseudo-randomised quasi-experiment, this study found that television advertising significantly increased both the likelihood and frequency of betting during live football games in the sample studied. This raises concerns about the adequacy of current advertising restrictions in the UK and suggests that a policy which restricts television gambling advertising around live football might be an effective part of a wider public health strategy to tackle gambling-related harms. Future studies should replicate this design, using larger, more representative samples, to inform policy.

## Highlights

• This study indicates that television gambling advertising increases betting during live football for men in England.
• These increases occur despite current industry-led restrictions on advertising during live sporting events.
• Results were highlighted using a novel, pseudo-randomised quasi-experiment.
• Restricting advertising during live sport may be a useful policy tool to reduce gambling behaviour and potential harm.

## 1 Introduction

Gambling is recognised as a public health issue, generating substantial health, social and economic costs estimated at approximately £1.05 to £1.77 billion annually in England alone (Bhattacharjee et al., 2023; Public Health England, 2023; Wardle et al., 2024). The harms extend well beyond the individual gambler to affect families, communities, and society, with negative consequences often persisting long after gambling ceases (Langham et al., 2016, Marionneau et al., 2023, Wardle et al., 2018).

In Great Britain, sports betting, particularly football, is one of the most prevalent forms of gambling (Gambling Commission, 2025) driven somewhat by technological advances over the last two decades. The rise of in-play and micro-betting has increased the speed and complexity of football betting, making it a more intensive and potentially harmful form of gambling (Torrance et al., 2024, Wardle et al., 2024). In the UK, men and those aged 18–44 (particularly 18–24) disproportionately represent the highest participation groups for sports betting, and they are also at greatest risk of gambling-related harm, as measured by the Problem Gambling Severity Index (PGSI) (Public Health England, 2023; Gambling Commission, 2025). Therefore, interventions that reduce gambling harm in this group are urgently needed.

One potential intervention is restricting gambling advertising, as implemented in several European countries (Wilson et al., 2024). Population-level approaches like these, as stated in Rose’s paradigm (1985), recognise that most harm arises from the many low- to moderate-risk gamblers, so even small behavioural changes across the population can yield substantial benefits. Gambling advertising spend in the UK has increased in recent years (Critchlow et al., 2022; Regulus Partners, 2018). Advertising is widespread, concentrated on sports, and often promotes complex, higher-risk bets (Deans et al., 2016, Newall et al., 2019, Torrance et al., 2021). Embedded advertising during live sports is particularly prevalent (Purves et al., 2020, Rossi et al., 2023, Torrance et al., 2023), and television remains a key source of exposure (Dunlop and Ballantyne, 2021, GambleAware., 2020, Syvertsen et al., 2022). Therefore, tighter regulation of gambling advertising during sport could reduce overall gambling participation and the incidence of gambling harm in the broader population.

Existing reviews indicate that gambling advertising is associated with increased gambling behaviour, particularly among those more vulnerable to harm (Bouguettaya et al., 2020, Killick and Griffiths, 2021, McGrane et al., 2025, McGrane et al., 2023). Advertisements have been cited as being the prime reason for opening a betting account (Dunlop & Ballantyne, 2021), a trigger to gamble (Binde, 2009, Grant and Kim, 2001, Hanss et al., 2015), prompting cravings and making it harder to abstain (Binde, 2009). Longitudinal research suggests that direct messaging by gambling companies is associated with betting intention, likelihood, and expenditure (Russell et al., 2018). Higher-risk individuals report that marketing prompts unplanned gambling spend (Wardle et al., 2022). However, despite methodological improvements in recent years, much of the evidence is based on observational or cross-sectional studies, which limits the ability to establish causality.

This study aims to fill an important evidence gap by using a quasi-experiment to estimate the impact of television gambling advertising on gambling behaviour amongst a higher-risk group of gamblers. It uses longitudinal betting surveys during the group stages of the 2022 FIFA World Cup held in Qatar. The study exploits the variation in gambling advertising between two broadcasters, Independent Television (ITV) and the British Broadcasting Corporation (BBC), to better identify causality in a real-world setting. Specifically, we aim to answer this research question:

*“Are a higher number of football bets placed during the game (‘in-play’) when a live game is televised on ITV (television gambling adverts) compared to BBC (no television gambling adverts)?”*

## 2 Methods

### 2.1 Methodological rationale

#### 2.1.1 Existing literature

The relationship between advertising and behaviour is complex; controlling for confounders is challenging. In observational studies, there are issues with endogeneity (e.g. reverse causality) which may bias estimates. In experimental studies (Di Censo et al., 2023, Houghton and Moss, 2020, Rockloff et al., 2019, Roderique-Davies et al., 2020), researchers can directly control for advertising exposure providing stronger internal validity. This permits demonstrations that the exposure caused the outcome but often lacks contextual factors that may be important for betting.

#### 2.1.2 Natural and quasi-experiments

Natural experiments are a type of quasi-experimental method that exploit an external (“exogenous”) variation in an explanatory variable to assess its impact on an outcome variable. These methods can address the limitations of observational and experimental studies, offering stronger causal inference in real-world settings when direct experimental manipulation is not feasible (de Vocht et al., 2021). Natural experiments have been used to evaluate policies in other areas (Adda et al., 2007, Jones et al., 2015, Wyper et al., 2023, Yau et al., 2022) but have been under-utilised in gambling research. The absence of large-scale sales data and the limited availability of survey data on gambling in the UK make retrospective policy analysis challenging. While frameworks that define and categorise quasi-experimental research exist (Craig et al., 2017, de Vocht et al., 2021, Remler and Van Ryzin, 2024), applying standard designs like Interrupted Time Series or Difference-in-Differences in gambling is often constrained by these limitations. Considering this, this study exploits naturally occurring variation in advertising exposure and collects primary data to assess this relationship.

#### 2.1.3 The quasi-experimental setup

The 2022 FIFA World Cup broadcasting rights in the UK were awarded to two free-to-air channels: ITV and the BBC. The BBC is primarily funded through a UK household licence fee, and it does not show commercial advertising. Conversely, ITV sells advertising slots around its programmes, including those to gambling operators. Advertisements for sports betting products are permitted around live sports broadcasts in the UK only within the pre- and post-match section, and not in the 5 min before kick-off, during half-time, or 5 min after the final whistle: known as the ‘whistle-to-whistle’ ban (Industry Group for Responsible Gambling, 2025). This creates a unique opportunity to use ITV as a proxy for television gambling advertising exposure.

#### 2.1.4 Assessing the quasi-experimental setup

**Fig. 1:**
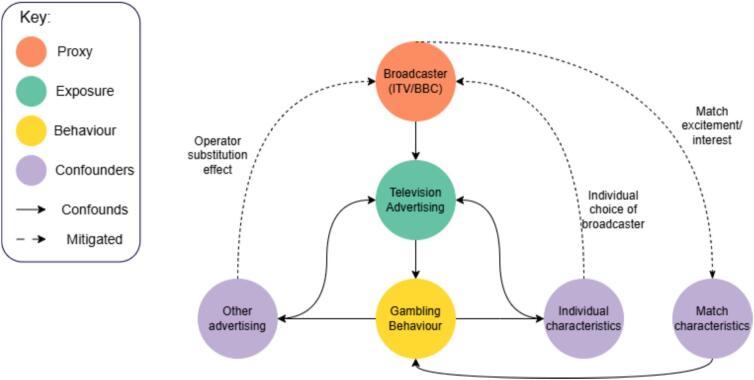
A causal loop diagram representing the quasi-experimental setup.

Fig. 1 presents a causal loop diagram outlining the quasi-experimental setup. Models based on self-reported exposure to advertising are prone to reverse causality since gambling behaviour correlates with individual characteristics and exposure to multiple types of advertising. Our design instead exploits variation in advertising between broadcasters. Because viewers cannot choose which broadcaster televises a given match, self-selection bias is minimised, and the externally assigned games help isolate the effect of television advertising on gambling behaviour.

The risk of substitution with other advertising forms is assumed to be limited. The tournament took place in Qatar where gambling is not legal. Only one pitch-side advert was recorded from an operator (‘Betano’) not yet available in the UK. While substitution through other channels (e.g. online or direct) is possible, the risk is assumed to be low. Television advertising delivers immediate and large-scale audience impacts, is among the most effective drivers of same-week profit, generates the highest overall advertising-attributable profit, and accounts for the largest share of web-traffic generation (Thinkbox, 2025). These advantages may be amplified in the context of major sporting events, where advertisers can align television spots with match schedules to influence viewer behaviour in tightly defined temporal windows.

Online and direct advertising can utilise more specific algorithmic targeting to individuals, but do not match the broad, immediate reach of television during mass-audience events. We therefore consider it more likely that operators shifted their television advertising to ITV’s post-match slots (ahead of BBC coverage) rather than redirecting spend to other formats, which may not offer comparable audience reach or return-on-investment.

Match allocation is assumed to be effectively random due to broadcaster competition and scheduling constraints, with descriptive results supporting this assumption (see section 3.2.1 and Appendix A). Random in-game events are unpredictable and challenging to measure and they should not systematically vary across broadcasters given our pseudo-random assumption. While we believe that this assumption is robust, to further strengthen the analysis we control for a range of measurable, structural match features that may influence betting behaviour (see section 2.9). We further reduce the risk that unobserved variation in match excitement or interest confounds our estimates by limiting our analysis to the group stages of the tournament. Summary statistics presented in Appendices A and D show that match characteristics and levels of engagement were consistent across broadcasters. Additionally, no teams received sponsorship from gambling companies during the tournament, and the official ITV tournament sponsors were unrelated to gambling, namely KFC and Google Pixel. These conditions strengthen our confidence in the model.

Given current data and practical constraints, this quasi-experimental design provides one of the most robust frameworks for estimating the causal impact of television gambling advertising that substantially mitigates concerns of reverse causality and confounding. This should strengthen confidence that any observed differences in gambling behaviour are driven by differences in gambling exposure between ITV and BBC, rather than by individual or external factors, in our sample.

#### 2.1.5 Necessary assumptions for causal inference

The key assumptions required for causal inference in this quasi-experimental setup are as follows:

1. Exogenous variation in television gambling advertising between broadcasters:
  • Supported by the ITV and BBC broadcasting arrangement.
2. No individual or operator selection of broadcaster:
  • Supported because match allocation occurs independently of viewers’ preferences and operators’ advertising slot choices.
3. No unobserved differences between matches televised on each broadcaster:
  • Supported by the pseudo-random allocation of matches.
4. No substitution effects to other forms of gambling advertising:
  • Partly supported by the pseudo-random allocation, and also by the absence of pitch-side advertising and the understanding that different advertising formats are not directly substitutable.

### 2.2 Participants

This study collected primary data on gambling behaviour from these individuals:

• **Sex**: males
• **Age**: 18 to 45
• **Gambling**: regular football gamblers (at least once in the last 12 months)
• **Location**: England
• **Other**: planning to watch some of the World Cup group-stage games.
• **Gambling**: no history of personal gambling problems (to minimise risk of harm).

Due to budget constraints, we recruited a purposive sample based on sex, age, location, and gambling behaviour. Males aged 18–45 were selected because these groups report higher gambling participation and risk of harm (Public Health England, 2023; Gambling Commission, 2025). Our sample covered the full spectrum of gambling behaviour, but higher-risk gamblers were overrepresented, so the results are exploratory and may not be generalisable to broader populations or other groups (e.g. women).

### 2.3 Sampling

This study uses purposive sampling, a method common in gambling advertising research (Browne et al., 2019, Hing et al., 2019, Lopez-Gonzalez and Griffiths, 2021, Russell et al., 2018). Since around half of adults in Great Britain do not gamble (Public Health England, 2023; Gambling Commission, 2025), purposive sampling helps focus on those most likely affected by gambling advertising, i.e. regular gamblers. Oversampling higher-risk gamblers ensures adequate representation, given their policy relevance and greater vulnerability (Hing et al., 2015). The study maximised sample size within the limits of available resources.

### 2.4 Recruitment

Participants were recruited via Prolific and completed surveys in Qualtrics. On 14th November 2022, an invitation was sent to 1,000 potential participants. After screening and consent, the 400 individuals with the highest football gambling frequency were purposively selected to ensure representation of higher-risk groups, following common gambling research practice (Hing et al., 2015). Gambling frequency was ranked using an adapted Health Survey for England measure, with only past-year gamblers eligible. Response time was used as a secondary sorting criterion. Participants received a detailed information sheet, provided digital consent, and were allowed to ask further questions about the study.

### 2.5 Surveys

On 17th November 2022, participants completed a baseline survey on demographics and gambling behaviour. From 21st November to 3rd December, they completed daily surveys released at 9am covering the previous match day (48 group stage matches over 13 days), closing after 48 h to minimise recall bias. Participants were asked to log into their betting accounts and manually enter the relevant information into the survey. We did not collect actual account records, as doing so was not feasible. Questions covered match viewing, betting activity, and details of any football bets placed (Appendix B Fig. B.1). A follow-up survey was issued on 5th December. Participants remained anonymised and were blinded to the study’s true purpose throughout.

### 2.6 Reimbursement

Reimbursement was up to £35, depending on survey completion, with payments ranging from £1.50 to £3.50 per survey. Each survey took 10 to 15 min totalling up to 3 h across 15 surveys, equating to £11.67 per hour. This amount was assumed to be fair to compensate participants without disproportionately incentivising participation. Participants were informed of the payment schedule before the study commenced.

### 2.7 Dependent variable

The dependent variable was the number of football bets placed during the game (‘in-play’), defined as those placed within a specific window (60, 30, 15, or 10 min) around a live game. Bets were operationalised by converting time-of-day betting data into minutes elapsed since midnight (Appendix B Fig. B.1), and all bets within the specified time window were included. Since there is no evidence for the optimal window for advertising effects, we conduct analyses across four windows based on the UK 'whistle-to-whistle' ban to assess whether effects varied across time.

Bet frequency was used as the primary variable instead of other outcome variables such as gambling expenditure, as it better reflects the likely causal mechanism of advertising: prompting additional bets. Though meaningful, expenditure may be confounded by factors such as income, and guidelines often recommend interpreting expenditure relative to income (Canadian Centre on Substance Use and Addiction, 2025; Victoria State Government, 2025; Young et al., 2024).

### 2.8 Independent variable

The key independent variable was a binary variable equal to 1 if the game was televised on ITV, and 0 if the game was televised on BBC.

### 2.9 Match level controls

To strengthen the analysis and control for non-advertising influences on betting, several match-level controls were selected based on data availability and their expected influence on betting:

Whether the participant self-reported watching the game
Whether the match was televised in the evening
Whether it was televised on a weekend (Saturday or Sunday)
Whether it was an England match
Whether it appeared in the Barb top-viewed programmes for the week
Whether the participant had already placed a bet on the match
A count of other ways the match was followed (e.g. online, radio, apps)
Match length (in minutes)
The absolute difference in FIFA ranking between the two teams (as of October 2022)

Match length was used instead of programme length to better reflect betting opportunities and avoid confounding. FIFA rankings proxied match predictability, and a ‘top viewed’ dummy variable was a proxy for match interest since viewing figures were unavailable. Odds data were not included due to the high complexity and dynamic nature of modern betting (varying substantially across time, operators, and bet types). Attempting to use a single odds variable would likely introduce measurement bias. Instead, structural match features predictive of betting behaviour are controlled for using the quasi-experimental design to strengthen causal inference. The FIFA ranking difference between teams, while not a perfect measure, is an appropriate proxy given data constraints.

### 2.10 Statistical analysis

This study used panel data methods with individual-level fixed effects, with the panel set at the individual (n = 365) and match (n = 48) level. Match-level fixed effects were excluded to avoid double-counting under the pseudo-random assumption and additional match-level controls. Given sampling limitations, but recognising the strengths of the design, the models estimate a treatment effect for this sample. A Poisson model was used for the count nature of the betting data, with a logistic model included for comparison. To ensure a balanced panel, only participants who completed all surveys were included in the main analysis. The analysis protocol and any adjustments were preregistered on Open Science Framework (https://osf.io/9uqt3/overview), and analyses were conducted in STATA 17. For the original and updated protocols and additional analyses, see chapter five of the associated open-access thesis (McGrane, 2025). The STROBE checklist is available in Appendix C.

### 2.11 Robustness checks

Negative Binomial models were run as a robustness check for potential overdispersion (Appendix F Table F.1), and both Akaike Information Criterion (AIC) and Bayesian Information Criterion (BIC) values were compared to identify the optimal statistical model (Appendix F Table F.2). Pairwise correlations are reported for all explanatory variables (Appendix F Table F.3).

### 2.12 Ethics

This study was approved by the University of Sheffield’s Ethics Review Procedure, as administered by the Sheffield Centre for Health and Related Research (SCHARR) [049521]. Informed consent was obtained digitally from all participants in the study.

## 3 Results

### 3.1 Recruitment

The 400 participants with the highest football gambling frequency were invited to the study. In total, 396 participants provided consent and completed the baseline survey before the study commenced. A total of 92 % of participants provided complete data, resulting in a final sample of 365 (Appendix B Fig. B.2).

### 3.2 Descriptive

#### 3.2.1 Differences in match excitement or interest

Appendix A summarises the characteristics of live matches between broadcasters. ITV and BBC covered a comparable number of England (ITV: n = 1, BBC: n = 2), top-viewed (ITV: n = 12, BBC: n = 11), weekend (ITV: n = 4, BBC: n = 5) and evening matches (ITV: n = 8, BBC: n = 9). Average match length (ITV: 101.7, BBC: 101.1 min) and FIFA ranking differences (ITV: 20.7, BBC: 21.2) were also comparable. Both covered debuts of top 10 FIFA-ranked teams (ITV: n = 6, BBC: n = 4), and team progression matches (ITV: n = 5, BBC: n = 9). ITV broadcasted key Spain/Germany advancement matches; BBC aired the Wales vs England match, the fourth most-watched UK programme of 2022 (Barb Audiences Ltd, 2023).

Formal statistical tests confirmed no statistically significant difference between broadcasters in terms of their match characteristics (Appendix A Table A.1) and therefore there is no clear reason to expect differences in betting between broadcasters based on the matches shown.

#### 3.2.2 Sociodemographic, gambling and other behavioural characteristics

Virtually the entire sample watched games on both ITV and BBC (see Appendix D). Participants' mean age was 33, they were predominantly White British and represented all English regions (Table 1). Life satisfaction was lower than the UK average (Office for National Statistics, 2023), though general and mental health were mostly good. On average, participants placed 10 bets and spent £78 per week (Table 2). A higher proportion of the sample showed medium or high risk of gambling harm compared to the UK general population, reflecting the purposive sampling.

**Table 1:** Sociodemographic characteristics of the quasi-experimental study.

*Variable*	*Detail*	*Sample*	
		*Mean (SD)*	*Range*
Age		33 (7)	[18, 45]

Life Satisfaction		6.4 (1.8)	[0, 10]

		*Frequency*	*Percentage*
Ethnicity			
	White British or Irish	285	78 %
	Mixed/ Multiple Ethnic Backgrounds	11	3 %
	Asian/Asian British	30	8 %
	Black/African/Caribbean/ Black British	18	5 %
	Other	21	6 %
Area of Residence			
	London	76	21 %
	South East	52	14 %
	North West	63	17 %
	East England	40	11 %
	East Midlands	32	9 %
	West Midlands	20	5 %
	North East	28	8 %
	Yorkshire & Humber	29	8 %
	South West	25	7 %

Employment			
	Employed	334	92 %
	Unemployed	31	8 %
Annual Income			
	£0-£9,999	17	5 %
	£10,000-£19,999	28	8 %
	£20,000-£29,999	88	24 %
	£30,000-£39,999	93	25 %
	£40,000-£49,999	66	18 %
	£50,000-£59,999	25	7 %
	£60,000-£69,999	18	5 %
	£70,000-£79,999	10	3 %
	>£79,999	20	5 %
General Health			
	Very Good	81	22 %
	Good	196	54 %
	Fair	82	22 %
	Bad	6	2 %
	Very Bad	0	0 %
Mental Health			
	Very Good	73	20 %
	Good	167	46 %
	Fair	106	29 %
	Bad	19	5 %
	Very Bad	0	0 %

**Table 2:** Gambling and other behavioural characteristics of the quasi-experimental study sample.

*Variable*	*Detail*	*Sample*	
		*Mean (SD)*	*Range*
Weekly Bets		10 (14)	*[1, 150]*
Weekly Spending on Bets		£77.88 (£155.34)	[£1, £1500]

Number of Accounts		6 (6.5)	*[1, 49]*

		*Frequency*	*Percentage*

Gambling Risk Level			
	No risk	95	26 %
	Lower Risk	128	35 %
	Medium Risk	103	28 %
	Higher Risk	39	11 %

Existing World Cup Bet			
	Yes	217	59 %
	No	148	41 %
Betting Alone			
	Almost always	120	33 %
	Most of the time	157	43 %
	Sometimes	85	23 %
	Never	3	1 %
Chosen Operator			
	Betfair	41	11 %
	Sky Bet	87	24 %
	Bet365	99	27 %
	Paddy Power	31	8 %
	Ladbrokes	24	7 %
	Coral	16	4 %
	Betfred	7	2 %
	LiveScore	2	1 %
	William Hill	49	13 %
	Other	9	2 %
Betting Types			
	Online betting on another sport/event	359	98 %
	National Lottery	241	66 %
	Online Games	198	54 %
	Horse Races	218	60 %
	Scratch Cards	173	47 %
	Sports events (bookmakers)	120	33 %
	Betting Exchange	154	42 %
	Fruit/Slot Machines	119	33 %
	Bingo	72	20 %
	Football Pools	51	14 %
	Virtual Gaming (bookmakers)	54	15 %
	Dog Races	38	10 %
	Table Games (Casino)	70	19 %
	Poker in a tournament	33	9 %
	Other events (bookmakers/phone)	22	6 %
Alcohol Risk Level			
	Low risk	224	61 %
	Increasing risk	109	30 %
	Higher risk	30	8 %
	Possible dependence	2	1 %

*Note: Gambling risk level measured using the Problem Gambling Severity Index (PGSI): 0 “no risk” 1–2 “low-risk” 3–7 “medium-risk” 8+ “higher-risk” (or ‘problem’ gambler); Alcohol risk level measured using the Alcohol Use Disorders Identification Test (AUDIT-C): 0–4 “low-risk” 5–7 “increasing-risk” 8–10 “higher-risk” 11–12 “possible dependence”. Participants could select multiple answers on the “betting types” question; One participant responded that they had 0 betting accounts with different companies, which we have assumed means they only hold one account with one company.

More in-game bets were placed during ITV matches than BBC matches (Appendix E Fig. E.1). The number of television gambling advertisements per game varied from 4 to 6, mostly shown during the pre-match build-up (Appendix E Fig. E.2). Advertising content ranged from simple branding to time-sensitive odds and match-specific promotions.

### 3.3 Poisson model

Frequency of betting on football was between 16 % and 24 % higher when games were televised on a channel with gambling advertising (ITV) compared to one without (BBC) [IRR: 1.16 – 1.24, p < 0.01] (Table 3). An IRR of 1.24 equates to roughly one additional bet per four baseline bets. All results were statistically significant, and coefficients increased as the windows around the game become narrower.

**Table 3:** Poisson regression model using the broadcaster (ITV) as the main explanatory variable.

	**Poisson 60**	**Poisson 30**	**Poisson 15**	**Poisson 10**
**ITV**	1.16^***^	1.16^***^	1.21^***^	1.24^***^
	**[1.07,1.25]**	**[1.05,1.29]**	**[1.07,1.38]**	**[1.07,1.43]**
Watch	1.09	1.10*	1.12*	1.15^**^
	[0.98,1.21]	[0.98,1.24]	[0.99,1.27]	[1.01,1.31]
Weekend	0.97	0.94	0.94	0.92
	[0.89,1.07]	[0.84,1.04]	[0.82,1.07]	[0.80,1.07]
Evening	0.60^***^	0.67^***^	0.57^***^	0.51^***^
	[0.53,0.69]	[0.55,0.81]	[0.45,0.73]	[0.39,0.68]
England	1.41^***^	1.39^***^	1.23*	1.09
	[1.24,1.61]	[1.17,1.65]	[0.99,1.53]	[0.86,1.38]
Top Views	0.80^***^	0.75^***^	0.77^***^	0.77^***^
	[0.74,0.87]	[0.68,0.82]	[0.70,0.85]	[0.69,0.85]
Match Length	1.01*	1.01	0.99	0.99
	[1.00,1.02]	[0.99,1.02]	[0.98,1.01]	[0.98,1.01]
Bet on Match	1.64^***^	1.47^***^	1.28^***^	1.21^**^
	[1.45,1.87]	[1.29,1.68]	[1.11,1.48]	[1.04,1.41]
Follow Match	1.08^**^	1.05	1.06	1.05
	[1.02,1.15]	[0.98,1.14]	[0.98,1.16]	[0.96,1.16]
Diff in FIFA Ranking	1.00	1.00	1.00^**^	1.00*
	[1.00,1.00]	[1.00,1.00]	[1.00,1.01]	[1.00,1.01]
Observations	16,656	16,320	15,936	15,792

Note: Key explanatory variable is a binary variable for the broadcaster (1 “ITV” 0 “BBC”); Poisson “n” is the window around the game e.g. Poisson 60 represents the 60-minute window; Coefficients are Incidence Rate Ratios (IRR) showing the change in the frequency of football bets placed ‘during the game’; Models use robust standard errors; Confidence intervals in parentheses; * p < 0.1, ^**^ p < 0.05, ^***^ p < 0.01.

Watching the game was positively associated with betting across all windows, although this was not always statistically significant. There was a reduced frequency of betting for games shown in the evening, and those with higher viewership. A greater frequency of football bets were placed on England games. There were no changes to bets placed during the game as countries grew closer in ranking, and therefore, the outcome might have been less certain.

### 3.4 Logistic model

Results for the Logistic regressions were similar; the explanatory variables showed similar signs and significance. Table 4 reports the change in the odds of placing a bet when matches were televised on ITV. Participants were 22 % to 33 % more likely to bet during games shown on a channel with gambling advertising (ITV) compared with one without (BBC) (Table 4). Table 5 presents the corresponding marginal effects, indicating a 2.1 to 6.7 percentage-point increase in the predicted probability of betting (in absolute terms) when advertising was present, although the estimate for the 60-minute window is only significant at the 10 % level.

**Table 4:** Logistic regression model using the broadcaster (ITV) as the main explanatory variable.

	**Logit 60**	**Logit 30**	**Logit 15**	**Logit 10**
**ITV**	1.22^***^	1.26^***^	1.31^***^	1.33^***^
	**[1.13,1.32]**	**[1.15,1.37]**	**[1.19,1.44]**	**[1.20,1.47]**
Watch	1.14^**^	1.10	1.12*	1.13*
	[1.02,1.26]	[0.98,1.24]	[0.99,1.27]	[0.99,1.29]
Weekend	0.97	0.96	0.96	0.98
	[0.88,1.07]	[0.86,1.07]	[0.86,1.09]	[0.86,1.10]
Evening	0.44^***^	0.48^***^	0.42^***^	0.37^***^
	[0.40,0.49]	[0.43,0.54]	[0.38,0.48]	[0.33,0.43]
England	1.90^***^	1.70^***^	1.53^***^	1.31^**^
	[1.61,2.25]	[1.41,2.04]	[1.24,1.89]	[1.04,1.65]
Top Views	0.80^***^	0.77^***^	0.80^***^	0.79^***^
	[0.73,0.88]	[0.70,0.86]	[0.72,0.90]	[0.71,0.89]
Match Length	1.02^***^	1.01*	1.00	1.00
	[1.01,1.03]	[1.00,1.03]	[0.99,1.02]	[0.98,1.02]
Bet on Match	1.99^***^	1.70^***^	1.43^***^	1.31^***^
	[1.79,2.20]	[1.52,1.91]	[1.27,1.62]	[1.15,1.49]
Follow Match	1.00	0.99	1.00	1.01
	[0.93,1.07]	[0.91,1.07]	[0.92,1.09]	[0.93,1.11]
Diff in FIFA Ranking	1.00	1.00	1.01^***^	1.01^***^
	[1.00,1.01]	[1.00,1.01]	[1.00,1.01]	[1.00,1.01]
Observations	16,656	16,320	15,936	15,792

Note: Key explanatory variable is a binary variable for the broadcaster (1 “ITV” 0 “BBC”); Logit “n” is the window around the game e.g. Logit 60 represents the 60-minute window; Coefficients are Odds Ratios (OR) showing changes in the likelihood of placing a football bet ‘during the game’; Confidence Intervals in parentheses; * p < 0.1, ^**^ p < 0.05, ^***^ p < 0.01.

**Table 5:** Marginal effects Logistic regression model using the broadcaster (ITV) as the main explanatory variable.

	Logit Marginal Effects 60	Logit Marginal Effects 30	Logit Marginal Effects 15	Logit Marginal Effects 10
**ITV**	0.021*	0.034^**^	0.063^***^	0.067^***^
	**[-0.001,0.043]**	**[0.001,0.067]**	**[0.039,0.088]**	**[0.044,0.090]**
Watch	0.013	0.014	0.027*	0.029*
	[-0.004,0.030]	[-0.007,0.035]	[-0.004,0.057]	[-0.001,0.060]
Weekend	−0.003	−0.006	−0.008	−0.006
	[-0.013,0.008]	[-0.022,0.011]	[-0.036,0.019]	[-0.034,0.023]
Evening	−0.085^**^	−0.108^**^	−0.202^***^	−0.230^***^
	[-0.168,-0.003]	[-0.202,-0.013]	[-0.241,-0.163]	[-0.261,-0.200]
England	0.067^**^	0.078^**^	0.101^***^	0.063^**^
	[0.002,0.132]	[0.007,0.149]	[0.050,0.151]	[0.009,0.117]
Top Views	−0.023*	−0.038^**^	−0.052^***^	−0.055^***^
	[-0.048,0.002]	[-0.075,-0.000]	[-0.079,-0.024]	[-0.081,-0.028]
Match Length	0.002^***^	0.002^***^	0.000	−0.000
	[0.001,0.003]	[0.002,0.003]	[-0.003,0.004]	[-0.004,0.004]
Bet on Match	0.072^**^	0.079^**^	0.085^***^	0.063^***^
	[0.002,0.141]	[0.008,0.149]	[0.053,0.116]	[0.033,0.093]
Follow Match	−0.000	−0.002	0.000	0.003
	[-0.008,0.007]	[-0.014,0.009]	[-0.019,0.020]	[-0.017,0.024]
Diff in FIFA Ranking	0.000	0.000	0.001^**^	0.001^***^
	[-0.000,0.001]	[-0.000,0.001]	[0.000,0.002]	[0.000,0.002]
Observations	16,656	16,320	15,936	15,792

Note: Key explanatory variable is a binary variable for the broadcaster (1 “ITV” 0 “BBC”); Logit marginal effects “n” is the window around the game e.g. Logit marginal effects 60 represents the 60-minute window; Coefficients are marginal effects showing the percentage point change in the probability of placing a football bet ‘during the game’; Confidence Intervals in parentheses; * p < 0.1, ^**^ p < 0.05, ^***^ p < 0.01.

### 3.5 Robustness check

Results were unchanged when using a Negative Binomial model, and both AIC and BIC values were lower for the Poisson models indicating a superior fit. Pairwise correlations between control variables were low and there were no concerns about multicollinearity (see Appendix F).

## 4 Discussion

This study examined how television gambling advertising influenced football betting among men in England during the 2022 World Cup. Using a pseudo-randomised quasi-experiment, we found that advertising significantly increased both the likelihood and frequency of betting across multiple time windows around the live game. These findings align with previous research suggesting a positive effect of advertising on gambling behaviour (Bouguettaya et al., 2020, Killick and Griffiths, 2021, McGrane et al., 2025, McGrane et al., 2023) and provide a stronger case for causality amongst this sample in a real-world context.

### 4.1 Strengths

This is the first study to use these methods in this area, uses a credible quasi-experimental design, and the findings align with prior research. It has high ecological validity by capturing real-world betting behaviour using participant-reported account data, which minimises recall bias. Further strengths include the preregistration of the statistical analysis plan, high compliance, and timely data collection.

### 4.2 Limitations

This study has several limitations, mainly stemming from data and evidence constraints in this research area. Generalisability is limited by purposive sampling and the recruitment panel (Pickering & Blaszczynski, 2021) alongside the exclusive use of male participants which could result in larger effects. The exclusion of individuals with possible gambling disorder (on ethical grounds) might have minimised the magnitude of the effects of advertising among the broader population. The results are strictly confined to gambling advertising during live sport, not extending to other gambling activities or advertising types. Potential for measurement and recall bias exists because betting behaviour was retrospectively self-reported, despite efforts to minimise this by limiting recall to 48 h and instructing participants to use their betting app data, though self-report bias is still a risk. Although we account for several match characteristics alongside a robust pseudo-randomisation assumption, some factors including pre-match programming interest could not be captured. Furthermore, a shift by operators toward alternative advertising formats (e.g. direct) around BBC programming to compensate for lost advertising opportunity could bias the effect size downward, though we assess this risk as low. A broadcaster proxy was used for advertising exposure, rather than self-reported match viewing. This was to overcome measurement issues, since time tuned into the match was unknown, and endogeneity, since the variable likely confounds with individual characteristics.

### 4.3 Policy implications

Despite a non-representative sample, the rigorous causal design of this study can provides relevant policy insights. Although current UK industry-led restrictions on television advertising have reduced the frequency of such advertising during the restricted (‘whistle-to-whistle’) period (McGrane et al., 2024, McGrane et al., 2024), these findings demonstrate a short-term behavioural response to television advertising, highlighting potential shortcomings of the restrictions, particularly for the higher-risk groups sampled. Advertising appears to raise overall gambling levels rather than just shifting market share between operators.

These findings are particularly relevant considering the upcoming 2026 World Cup. Since the 2022 tournament, there have been no statutory or self-regulatory changes to gambling advertising scheduling laws in the UK. Existing evidence lends support to the total consumption theory of gambling, which links higher average levels of gambling to greater population-level harm (Kesaite et al., 2023). In this context, increased gambling among already high-risk groups may increase overall population harm, which has important public health policy implications.

## 5 Conclusion

This study explored the impact of television gambling advertising on the betting behaviour of men in England using a novel pseudo-randomised quasi-experimental design. Results indicate that the frequency of betting on football is 16 % to 24 % higher, and the probability of betting on football is 22 % to 33 % higher, when a game contains television gambling advertising compared to when it does not, amongst this sample. These results suggest that a policy which restricts television advertising of gambling around live football might be an effective component of a broader public health strategy to tackle gambling-related harms, given the findings highlighted in this population group. However, future studies should replicate this design using more representative samples, potentially using verified betting data, and using mixed-gender samples, to further inform policy.

## CRediT authorship contribution statement

**Ellen McGrane:** Writing – review & editing, Writing – original draft, Visualization, Validation, Software, Resources, Project administration, Methodology, Investigation, Funding acquisition, Formal analysis, Data curation, Conceptualization. **Robert Pryce:** Writing – review & editing, Writing – original draft, Visualization, Supervision, Software, Resources, Project administration, Methodology, Investigation, Formal analysis, Data curation, Conceptualization. **Matt Field:** Writing – review & editing, Writing – original draft, Visualization, Supervision, Resources, Project administration, Methodology, Investigation. **Luke Wilson:** Writing – review & editing, Writing – original draft, Methodology, Formal analysis. **Elizabeth Goyder:** Writing – review & editing, Writing – original draft, Visualization, Supervision, Resources, Project administration, Methodology, Investigation, Conceptualization.

## Funding

This work was funded by a Wellcome Trust grant [224852/Z/21/Z].

## Declaration of competing interest

The authors declare the following financial interests/personal relationships which may be considered as potential competing interests: MF has received grants from the Medical Research Council and the Academic Forum for the Study of Gambling within the past 36 months. MF is also a trustee of the Society for the Study of Addiction. The remaining authors have no conflicts of interest to declare.

## Data Availability

Data will be made available on request.

## References

[b0005] Adda J., Berlinski S., Machin S. (2007). Short-run economic effects of the Scottish smoking ban. International Journal of Epidemiology.

[b0010] Barb Audiences Ltd. (2023, January). *What people watch: Viewing in 2022*. https://www.barb.co.uk/insight-parent/insight-what-people-watch/what-people-watch-viewing-in-2022/.

[b0015] Bhattacharjee, A., Dolton, P., Mosley, M., & Pabst, A. (2023). *The fiscal costs and benefits of problem gambling: Towards better estimates*. National Institute of Economic and Social Research. https://niesr.ac.uk/wp-content/uploads/2023/04/The-Fiscal-Costs-and-Benefits-of-Problem-Gambling-1.pdf.

[b0020] Binde P. (2009). Exploring the impact of gambling advertising: An interview study of problem gamblers. International Journal of Mental Health and Addiction.

[b0025] Bouguettaya A., Lynott D., Carter A., Zerhouni O., Meyer S., Ladegaard I., Gardner J., O’Brien K.S. (2020). The relationship between gambling advertising and gambling attitudes, intentions and behaviours: A critical and meta-analytic review. Current Opinion in Behavioral Sciences.

[b0030] Browne M., Hing N., Russell A.M.T., Thomas A., Jenkinson R. (2019). The impact of exposure to wagering advertisements and inducements on intended and actual betting expenditure: An ecological momentary assessment study. Journal of Behavioral Addictions.

[b0035] Canadian Centre on Substance Use and Addiction. (2025). *The lower-risk gambling guidelines*. https://gamblingguidelines.ca/.

[b0040] Craig P., Katikireddi S.V., Leyland A., Popham F. (2017). Natural experiments: An overview of methods, approaches, and contributions to public health intervention research. Annual Review of Public Health.

[b0045] Critchlow N., Hunt K., Wardle H., Stead M. (2022). Expenditure on paid-for gambling advertising during the national COVID-19 ‘lockdowns’: An observational study of media monitoring data from the United Kingdom. Journal of Gambling Studies.

[b0050] de Vocht F., Katikireddi S.V., McQuire C., Tilling K., Hickman M., Craig P. (2021). Conceptualising natural and quasi experiments in public health. BMC Medical Research Methodology.

[b0055] Deans E.G., Thomas S.L., Daube M., Derevensky J., Gordon R. (2016). Creating symbolic cultures of consumption: An analysis of the content of sports wagering advertisements in Australia. BMC Public Health.

[b0060] Di Censo G., Delfabbro P., King D.L. (2023). Young people’s perceptions of the effects and value of sports betting inducements. International Journal of Mental Health and Addiction.

[b0065] Dunlop P., Ballantyne E.E.F. (2021). Effective and responsible marketing of online sports gambling to young adults in the UK. SN Business & Economics.

[b0070] GambleAware. (2020). *The effect of gambling marketing and advertising on children, young people and vulnerable adults*. https://www.ipsos.com/en-uk/effect-gambling-advertising-children-young-people-and-vulnerable-adults.

[b0075] Gambling Commission. (2025). *Gambling survey for Great Britain: Annual report year 2 (2024)*. https://www.gamblingcommission.gov.uk/statistics-and-research/publication/statistics-on-gambling-participation-annual-report-year-2-2024-official.

[b0080] Grant J.E., Kim S.W. (2001). Demographic and clinical features of 131 adult pathological gamblers. The Journal of Clinical Psychiatry.

[b0085] Hanss D., Mentzoni R.A., Griffiths M.D., Pallesen S. (2015). The impact of gambling advertising: Problem gamblers report stronger impacts on involvement, knowledge, and awareness than recreational gamblers. Psychology of Addictive Behaviors.

[b0090] Hing N., Lamont M., Vitartas P., Fink E. (2015). Sports-embedded gambling promotions: A study of exposure, sports betting intention and problem gambling amongst adults. International Journal of Mental Health and Addiction.

[b0095] Hing N., Russell A.M.T., Thomas A., Jenkinson R. (2019). Wagering ddvertisements and inducements: Exposure and perceived influence on betting behaviour. Journal of Gambling Studies.

[b0100] Houghton S., Moss M. (2020). Comparing football bettors’ response to social media marketing differing in bet complexity and account type – an experimental study. Journal of Behavioral Addictions.

[b0105] Industry Group for Responsible Gambling. (2025). *Gambling industry code for socially responsible advertising* (7th ed.). Betting & Gaming Council. https://bettingandgamingcouncil.com/members/igrg.

[b0110] Jones A.M., Laporte A., Rice N., Zucchelli E. (2015). Do public smoking bans have an impact on active smoking?. Evidence from the UK. Health Economics.

[b0115] Kesaite V., Wardle H., Rossow I. (2023). Gambling consumption and harm: A systematic review of the evidence. Addiction Research and Theory.

[b0120] Killick E., Griffiths M.D. (2021). Impact of sports betting advertising on gambling behavior: A systematic review. Addicta: The Turkish Journal on Addictions.

[b0125] Langham E., Thorne H., Browne M., Donaldson P., Rose J., Rockloff M. (2016). Understanding gambling related harm: A proposed definition, conceptual framework, and taxonomy of harms. BMC Public Health.

[b0130] Lopez-Gonzalez H., Griffiths M.D. (2021). Brand knowledge, similarity to story characters and perceived influence of gambling advertising among Spanish sports bettors: A survey study. International Journal of Mental Health and Addiction.

[b0135] Marionneau V., Egerer M., Raisamo S. (2023). Frameworks of gambling harms: A comparative review and synthesis. Addiction Research and Theory.

[b0140] McGrane, E. (2025). What is the impact of restrictions on television gambling advertising during live sport? An investigation using quasi-experimental and econometric methods [Unpublished doctoral dissertation]. The University of Sheffield. https://etheses.whiterose.ac.uk/id/eprint/37323/.

[b0145] McGrane E., Pryce R., Field M., Goyder E. (2024). The association between the ‘whistle-to-whistle’ ban and the presence of gambling advertising on UK television. Addiction Research & Theory.

[b0150] McGrane E., Pryce R., Field M., Gu S., Moore E.C., Goyder E. (2025). What is the impact of sports-related gambling advertising on gambling behaviour? A systematic review. Addiction.

[b0155] McGrane E., Pryce R., Wilson L., Field M., Goyder E. (2024). How did the ‘whistle-to-whistle’ ban affect gambling advertising on TV? a live football matching study. Addiction Research & Theory.

[b0160] McGrane E., Wardle H., Clowes M., Blank L., Pryce R., Field M., Sharpe C., Goyder E. (2023). What is the evidence that advertising policies could have an impact on gambling-related harms? a systematic umbrella review of the literature. Public Health.

[b0165] Newall P.W.S., Moodie C., Reith G., Stead M., Critchlow N., Morgan A., Dobbie F. (2019). Gambling marketing from 2014 to 2018: A literature review. Current Addiction Reports.

[b0170] Office for National Statistics. (2023). *Personal well-being in the UK: April 2022 to March 2023*. https://www.ons.gov.uk/peoplepopulationandcommunity/wellbeing/bulletins/measuringnationalwellbeing/april2022tomarch2023.

[b0175] Pickering D., Blaszczynski A. (2021). Paid online convenience samples in gambling studies: Questionable data quality. International Gambling Studies.

[b0180] Public Health England. (2023). *Gambling-related harms: Evidence review*. Department of Health and Social Care. https://www.gov.uk/government/publications/gambling-related-harms-evidence-review.

[b0185] Purves R.I., Critchlow N., Morgan A., Stead M., Dobbie F. (2020). Examining the frequency and nature of gambling marketing in televised broadcasts of professional sporting events in the United Kingdom. Public Health.

[b0190] Regulus Partners. (2018). *Gambling advertising and marketing spend in Great Britain, 2014–17*. GambleAware. https://www.gambleaware.org/media/ym5dba3l/2018-11-24-rp-ga-gb-marketing-spend-infographic-final.pdf.

[b0195] Remler D.K., Van Ryzin G.G. (2024). Control, exogeneity, and directness: Understanding and designing quasi- and natural experiments. American Journal of Evaluation.

[b0200] Rockloff M.J., Browne M., Russell A.M.T., Hing N., Greer N. (2019). Sports betting incentives encourage gamblers to select the long odds: An experimental investigation using monetary rewards. Journal of Behavioral Addictions.

[b0205] Roderique-Davies G., Torrance J., Bhairon T., Cousins A., John B. (2020). Embedded gambling promotion in football: An explorative study of cue-exposure and urge to gamble. Journal of Gambling Studies.

[b0210] Rose G. (1985). Sick individuals and sick populations. International Journal of Epidemiology.

[b0215] Rossi, R., Wheaton, J., Moxey, M., & Tozzi, E. (2023). New season, more self-regulation, more marketing: The prevalence of gambling adverts during the opening weekend of the English Premier League 2023/24. University of Bristol. https://www.bristol.ac.uk/media-library/sites/business-school/documents/BRISTOL-UNI-GAMBLING-Report2023-2.pdf.

[b0220] Russell A.M.T., Hing N., Browne M., Rawat V. (2018). Are direct messages (texts and emails) from wagering operators associated with betting intention and behavior? an ecological momentary assessment study. Journal of Behavioral Addictions.

[b0225] Syvertsen A., Erevik E.K., Hanss D., Mentzoni R.A., Pallesen S. (2022). Relationships between exposure to different gambling advertising types, advertising impact and problem gambling. Journal of Gambling Studies..

[b0230] Thinkbox. (2025). *Why TV?* https://www.thinkbox.tv/why-tv.

[b0235] Torrance J., Heath C., Andrade M., Newall P. (2023). Gambling, cryptocurrency, and financial trading app marketing in English Premier League football: A frequency analysis of in-game logos. Journal of Behavioral Addictions.

[b0240] Torrance J., John B., Greville J., O’Hanrahan M., Davies N., Roderique-Davies G. (2021). Emergent gambling advertising; a rapid review of marketing content, delivery and structural features. BMC Public Health.

[b0245] Torrance J., O’Hanrahan M., Carroll J., Newall P. (2024). The structural characteristics of online sports betting: A scoping review of current product features and utility patents as indicators of potential future developments. Addiction Research & Theory.

[b0250] Victoria State Government. (2025). *Lower risk gambling guidelines*. Gambler’s Help. https://gamblershelp.com.au/how-often-you-gamble-matters/lower-risk-gambling-guidelines/.

[b0255] Wardle H., Critchlow N., Brown A., Donnachie C., Kolesnikov A., Hunt K. (2022). The association between gambling marketing and unplanned gambling spend: Synthesised findings from two online cross-sectional surveys. Addictive Behaviors.

[b0260] Wardle H., Degenhardt L., Marionneau V., Reith G., Livingstone C., Sparrow M., Tran L.T., Biggar B., Bunn C., Farrell M., Kesaite V., Poznyak V., Quan J., Rehm J., Rintoul A., Sharma M., Shiffman J., Siste K., Saxena S. (2024). The Lancet Public Health Commission on gambling. The Lancet Public Health.

[b0265] Wardle, H., Reith, G., Best, D., McDaid, D., & Platt, S. (2018). *Measuring gambling-related harms: A framework for action*. The London School of Economics and Political Science. .

[b0270] Wilson, J., Rossi, R., Bransden, N., Amos, M., & Sakis, P. (2024). *Drivers of gambling marketing restrictions: An international comparison*. Ipsos; GambleAware; University of Bristol. https://www.gambleaware.org/media/bjqev1hx/drivers-of-gambling-marketing-restrictions-an-international-comparison-v3.pdf.

[b0275] Wyper G.M.A., Mackay D.F., Fraser C., Lewsey J., Robinson M., Beeston C., Giles L. (2023). Evaluating the impact of alcohol minimum unit pricing on deaths and hospitalisations in Scotland: A controlled interrupted time series study. The Lancet.

[b0280] Yau A., Berger N., Law C., Cornelsen L., Greener R., Adams J., Boyland E.J., Burgoine T., de Vocht F., Egan M., Er V., Lake A.A., Lock K., Mytton O., Petticrew M., Thompson C., White M., Cummins S. (2022). Changes in household food and drink purchases following restrictions on the advertisement of high fat, salt, and sugar products across the Transport for London network: A controlled interrupted time series analysis. PLoS Medicine.

[b0285] Young M.M., Hodgins D.C., Currie S.R., Brunelle N., Dufour M., Flores-Pajot M.-C., Nadeau L. (2024). Not too much, not too often, and not too many: The results of the first large-scale, international project to develop lower-risk gambling guidelines. International Journal of Mental Health and Addiction.

