# Supplementary material for: The effect of television advertising on gambling behaviour: a quasi-experimental study during the 2022 Qatar FIFA World Cup
Source: Addict Behav Rep. 2026 Jan 13;23:100666. doi: 10.1016/j.abrep.2026.100666 (PMC12854864; doi:10.1016/j.abrep.2026.100666)
Supplement: Supplementary Data 1 [file mmc1.docx]

**Appendices**

Contents

[Appendix A: Match Characteristics 3](#_Toc215756511)

[Appendix A Table A.1: Details of match characteristics between BBC and ITV broadcasts 4](#_Toc215756512)

[Appendix A Table A.2: Details of knock-out matches 5](#_Toc215756513)

[Appendix B: Survey and Recruitment 7](#_Toc215756514)

[Appendix B Figure B.1: An example of the daily betting survey 7](#_Toc215756515)

[Appendix B Figure B.2: Recruitment Flow Chart 8](#_Toc215756516)

[Appendix C: STROBE Checklist for Observational Studies 10](#_Toc215756517)

[Appendix D: Characteristics of Participants by Broadcaster 15](#_Toc215756518)

[Appendix D Table D.1: Sociodemographic characteristics of the sample by broadcaster 17](#_Toc215756519)

[Appendix D Figure D. 2: Gambling and other behavioural characteristics of the sample by broadcaster 19](#_Toc215756520)

[Appendix E: Betting and Advertising During Live Games 21](#_Toc215756521)

[Appendix E Figure E.2: The frequency of television advertisements by match (60-minute window) 21](#_Toc215756522)

[Appendix E Figure E.1: The frequency of bets placed during the game by match and broadcaster (60-minute window) 21](#_Toc215756523)

[Appendix F: Robustness Checks 24](#_Toc215756524)

[Appendix F Table F. 1: Negative Binomial models 24](#_Toc215756525)

[Appendix F Table F.2: Akaike’s Information Criterion (AIC) and Bayesian Information Criterion (BIC) for Poisson and Negative Binomial models 25](#_Toc215756526)

[Appendix F Table F.3: Pairwise correlations between explanatory variables 25](#_Toc215756527)

**Appendix A:**

**Match Characteristics**

## Appendix A: Match Characteristics

| Measure | Match Excitement or Interest | ITV | BBC | P-value |
| --- | --- | --- | --- | --- |
|  | England games | 1 | 2 | 0.55 |
|  | One of the top viewed programmes of that week | 12 | 11 | 0.78 |
|  | Weekend games (Saturday/Sunday) | 4 | 5 | 0.51 |
|  | Evening games (7pm) | 8 | 9 | 1.00 |
|  | First game played by one of the top 10 teams in the October 2022 FIFA rankings | 6 | 4 | 0.48 |
|  | Determined progression to the next stage of the tournament | 5 | 9 | 0.20 |
|  | Top 50 most highly viewed broadcasts of 2022 | 0 | 1 | 0.31 |
|  | Length of the match (minutes including added time) | 101.70 | 101.10 | 0.38 |
|  | Difference in the October 2022 FIFA rankings between the teams playing | 20.70 | 21.20 | 0.71 |
|  | Self-reported watching | 0.57 | 0.56 | 0.18 |

Appendix A Table A.1: Details of match characteristics between BBC and ITV broadcasts; *Sources - BARB* [*https://www.barb.co.uk/viewing-data/most-viewed-programmes/*](https://www.barb.co.uk/viewing-data/most-viewed-programmes/)*;* [*https://www.barb.co.uk/insight-parent/insight-what-people-watch/what-people-watch-viewing-in-2022/*](https://www.barb.co.uk/insight-parent/insight-what-people-watch/what-people-watch-viewing-in-2022/)*; Sporting News* [*https://www.sportingnews.com/uk/football/news/teams-out-world-cup-2022-list-nations-eliminated-fifa-2022/cmk6aexisveysdxidiq84baf*](https://www.sportingnews.com/uk/football/news/teams-out-world-cup-2022-list-nations-eliminated-fifa-2022/cmk6aexisveysdxidiq84baf)*;* [*https://www.sportingnews.com/us/soccer/news/world-cup-standings-2022-table-live-updated-group-qatar/lv5qodvbdsecrwf0gwelqzn0*](https://www.sportingnews.com/us/soccer/news/world-cup-standings-2022-table-live-updated-group-qatar/lv5qodvbdsecrwf0gwelqzn0); ‘p-value’ is from either a Chi-squared test or in the case of continuous variables a Mann-Whitney U-test where H0: there are no statistically significant differences between ITV and BBC, in all cases we fail to reject the null based on standard levels of statistical significance and conclude that there are no statistically significant differences between match characteristics across broadcasters.

| **Match Excitement/Interest** | **ITV** | **BBC** |
| --- | --- | --- |
| *Determined knock-out* | **Ecuador Vs Senegal:** Ecuador lose against Senegal and are knocked out | **Iran Vs USA:** Iran lose against USA and are knocked out |
|  | **Japan Vs Spain:** Spain knock out Germany on goals scored. | **Wales Vs England:** Wales lose against England and are knocked out |
|  | **Costa Rica Vs Germany:** Costa Rica knocked out and Germany knocked out on goal difference with Spain. | **France Vs Tunisia:** France win against Tunisia and Tunisia knocked out on goal difference with Australia. |
|  | **Serbia Vs Switzerland:** Switzerland go through, and Serbia knocked out | **Australia Vs Denmark:** Australia go through on goal difference to Tunisia and Denmark are knocked out |
|  | **Cameroon vs Brazil:** Switzerland win against Serbia and knock out Cameroon. | **Poland Vs Argentina:** Poland go through on goal difference and knock out Mexico |
|  |  | **Saudi Arabia Vs Mexico:** Saudi Arabia knocked out and Mexico knocked out on goal difference to Poland. |
|  |  | **Croatia Vs Belgium:** A draw between these teams saw Croatia kicked out. |
|  |  | **South Korea Vs Portugal:** South Korea kick Uruguay out on goals scored. |
|  |  | **Ghana Vs Uruguay:** Ghana lose and are kicked out, Uruguay kicked out on goals scored compared to South Korea. |

Appendix A Table A.2: Details of knock-out matches; *Sources -* [*https://www.sportingnews.com/uk/football/news/teams-out-world-cup-2022-list-nations-eliminated-fifa-2022/cmk6aexisveysdxidiq84baf*](https://www.sportingnews.com/uk/football/news/teams-out-world-cup-2022-list-nations-eliminated-fifa-2022/cmk6aexisveysdxidiq84baf)*;* [*https://www.sportingnews.com/us/soccer/news/world-cup-standings-2022-table-live-updated-group-qatar/lv5qodvbdsecrwf0gwelqzn0*](https://www.sportingnews.com/us/soccer/news/world-cup-standings-2022-table-live-updated-group-qatar/lv5qodvbdsecrwf0gwelqzn0)

**Appendix B:**

**Survey and Recruitment**

##
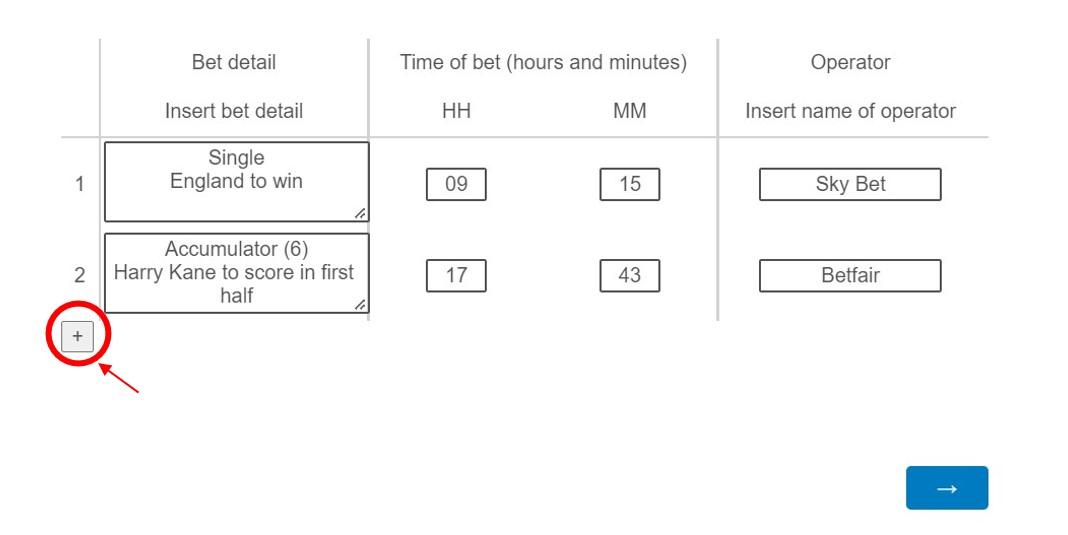
Appendix B: Survey and Recruitment

### Appendix B Figure B.1: An example of the daily betting survey


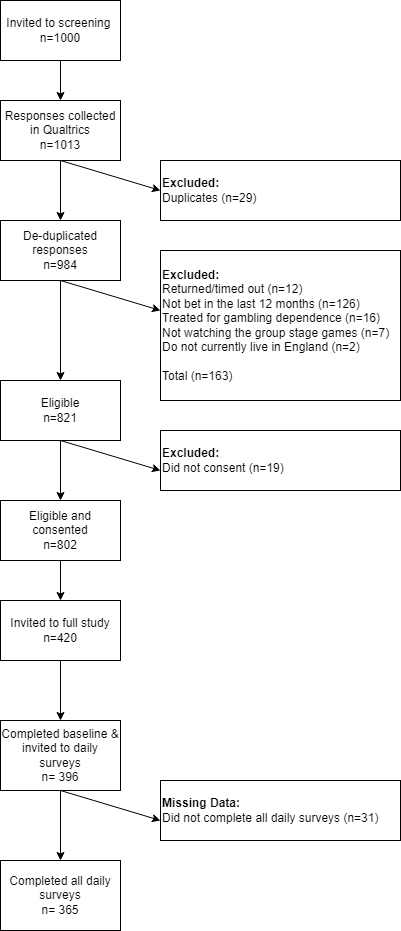


### Appendix B Figure B.2: Recruitment Flow Chart

**Appendix C:**

**STROBE Checklist**

## Appendix C: STROBE Checklist for Observational Studies

|  | **Item No** | **Recommendation** | **Checked** |
| --- | --- | --- | --- |
| **Title and abstract** | 1 | (*a*) Indicate the study’s design with a commonly used term in the title or the abstract | YES  Page 1 |
|  |  | (*b*) Provide in the abstract an informative and balanced summary of what was done and what was found | YES  Page 1 |
| **Introduction** | | |  |
| Background/rationale | 2 | Explain the scientific background and rationale for the investigation being reported | YES  Pages 2 - 3 |
| Objectives | 3 | State specific objectives, including any prespecified hypotheses | YES  Page 3 |
| **Methods** | | |  |
| Study design | 4 | Present key elements of study design early in the paper | YES  Pages 3-6 |
| Setting | 5 | Describe the setting, locations, and relevant dates, including periods of recruitment, exposure, follow-up, and data collection | YES  Pages 6-7 |
| Participants | 6 | (*a*) *Cohort study*—Give the eligibility criteria, and the sources and methods of selection of participants. Describe methods of follow-up  *Case-control study*—Give the eligibility criteria, and the sources and methods of case ascertainment and control selection. Give the rationale for the choice of cases and controls  *Cross-sectional study*—Give the eligibility criteria, and the sources and methods of selection of participants | YES  Pages 6-7 |
|  |  | (*b*) *Cohort study*—For matched studies, give matching criteria and number of exposed and unexposed  *Case-control study*—For matched studies, give matching criteria and the number of controls per case | N/A |
| Variables | 7 | Clearly define all outcomes, exposures, predictors, potential confounders, and effect modifiers. Give diagnostic criteria, if applicable | YES  Pages 3-6  Pages 7-8 |
| Data sources/ measurement | 8* | For each variable of interest, give sources of data and details of methods of assessment (measurement). Describe comparability of assessment methods if there is more than one group | YES  Pages 7-8 |
| Bias | 9 | Describe any efforts to address potential sources of bias | YES  Pages 3-9 |
| Study size | 10 | Explain how the study size was arrived at | YES  Page 6 |
| Quantitative variables | 11 | Explain how quantitative variables were handled in the analyses. If applicable, describe which groupings were chosen and why | YES  Page 7-8 |
| Statistical methods | 12 | (*a*) Describe all statistical methods, including those used to control for confounding | YES  Pages 7-9 |
|  |  | (*b*) Describe any methods used to examine subgroups and interactions | N/A |
|  |  | (*c*) Explain how missing data were addressed | YES  Page 8 |
|  |  | (*d*) *Cohort study*—If applicable, explain how loss to follow-up was addressed  *Case-control study*—If applicable, explain how matching of cases and controls was addressed  *Cross-sectional study*—If applicable, describe analytical methods taking account of sampling strategy | N/A |
|  |  | (*e*) Describe any sensitivity analyses | YES  Page 9 link to open access thesis |

**Results**

| Participants | 13* | (a) Report numbers of individuals at each stage of study—eg numbers potentially eligible, examined for eligibility, confirmed eligible, included in the study, completing follow-up, and analysed | YES  Appendix B |
| --- | --- | --- | --- |
|  |  | (b) Give reasons for non-participation at each stage | YES  Appendix B |
|  |  | (c) Consider use of a flow diagram | YES  Appendix B |
| Descriptive data | 14* | (a) Give characteristics of study participants (eg demographic, clinical, social) and information on exposures and potential confounders | YES  Pages 10-13  Appendix D |
|  |  | (b) Indicate number of participants with missing data for each variable of interest | N/A |
|  |  | (c) *Cohort study*—Summarise follow-up time (eg, average and total amount) | N/A |
| Outcome data | 15* | *Cohort study*—Report numbers of outcome events or summary measures over time | YES  Appendix E |
|  |  | *Case-control study—*Report numbers in each exposure category, or summary measures of exposure | N/A |
|  |  | *Cross-sectional study—*Report numbers of outcome events or summary measures | N/A |
| Main results | 16 | (*a*) Give unadjusted estimates and, if applicable, confounder-adjusted estimates and their precision (eg, 95% confidence interval). Make clear which confounders were adjusted for and why they were included | YES  Page 15-16 |
|  |  | (*b*) Report category boundaries when continuous variables were categorized | N/A |
|  |  | (*c*) If relevant, consider translating estimates of relative risk into absolute risk for a meaningful time period | N/A |
| Other analyses | 17 | Report other analyses done—eg analyses of subgroups and interactions, and sensitivity analyses | YES  YES  Page 9 link to open access thesis |
| **Discussion** | | |  |
| Key results | 18 | Summarise key results with reference to study objectives | YES  Page 16 |
| Limitations | 19 | Discuss limitations of the study, taking into account sources of potential bias or imprecision. Discuss both direction and magnitude of any potential bias | YES  Page 17 |
| Interpretation | 20 | Give a cautious overall interpretation of results considering objectives, limitations, multiplicity of analyses, results from similar studies, and other relevant evidence | YES  Pages 16-17 |
| Generalisability | 21 | Discuss the generalisability (external validity) of the study results | YES  Page 17 |
| **Other information** | | |  |
| Funding | 22 | Give the source of funding and the role of the funders for the present study and, if applicable, for the original study on which the present article is based | YES  Title Page |

**Appendix D: Participant Characteristics (by broadcaster)**

## Appendix D: Characteristics of Participants by Broadcaster

| *Variable* | *Detail* | *Total Sample* | | *Watch ITV* | | *Watch BBC* | |
| --- | --- | --- | --- | --- | --- | --- | --- |
|  |  | *Mean (SD)* | *Range* | *Mean (SD)* | *Range* | *Mean (SD)* | *Range* |
| Age |  | 33 (7) | [18, 45] | 33 (7) | [18, 45] | 33 (7) | [18, 45] |
|  |  |  |  |  |  |  |  |
| Life Satisfaction |  | 6.4 (1.8) | [0, 10] | 6.4 (1.8) | [0, 10] | 6.4 (1.8) | [0, 10] |
|  |  |  |  |  |  |  |  |
|  |  |  |  |  |  |  |  |
|  |  | *Frequency* | *Percentage* | *Frequency* | *Percentage* | *Frequency* | *Percentage* |
| Ethnicity |  |  |  |  |  |  |  |
|  | White British or Irish | 285 | 78% | 282 | 78% | 285 | 78% |
|  | Mixed/ Multiple Ethnic Backgrounds | 11 | 3% | 11 | 3% | 11 | 3% |
|  | Asian/Asian British | 30 | 8% | 30 | 8% | 30 | 8% |
|  | Black/African/Caribbean/ Black British | 18 | 5% | 18 | 5% | 18 | 5% |
|  | Other | 21 | 6% | 21 | 6% | 21 | 6% |
| Area of Residence |  |  |  |  |  |  |  |
|  | London | 76 | 21% | 75 | 21% | 76 | 21% |
|  | South East | 52 | 14% | 52 | 14% | 52 | 14% |
|  | North West | 63 | 17% | 63 | 17% | 63 | 17% |
|  | East England | 40 | 11% | 40 | 11% | 40 | 11% |
|  | East Midlands | 32 | 9% | 32 | 9% | 32 | 9% |
|  | West Midlands | 20 | 5% | 19 | 5% | 20 | 5% |
|  | North East | 28 | 8% | 28 | 8% | 28 | 8% |
|  | Yorkshire & Humber | 29 | 8% | 29 | 8% | 29 | 8% |
|  | South West | 25 | 7% | 24 | 7% | 25 | 7% |
|  |  |  |  |  |  |  |  |
| Employment |  |  |  |  |  |  |  |
|  | Employed | 334 | 92% | 331 | 91% | 334 | 92% |
|  | Unemployed | 31 | 8% | 31 | 9% | 31 | 8% |
| Annual Income |  |  |  |  |  |  |  |
|  | £0-£9,999 | 17 | 5% | 17 | 5% | 17 | 5% |
|  | £10,000-£19,999 | 28 | 8% | 28 | 8% | 28 | 8% |
|  | £20,000-£29,999 | 88 | 24% | 88 | 24% | 88 | 24% |
|  | £30,000-£39,999 | 93 | 25% | 93 | 26% | 93 | 25% |
|  | £40,000-£49,999 | 66 | 18% | 65 | 18% | 66 | 18% |
|  | £50,000-£59,999 | 25 | 7% | 24 | 7% | 25 | 7% |
|  | £60,000-£69,999 | 18 | 5% | 18 | 5% | 18 | 5% |
|  | £70,000-£79,999 | 10 | 3% | 9 | 2% | 10 | 3% |
|  | >£79,999 | 20 | 5% | 20 | 5% | 20 | 5% |
| General Health |  |  |  |  |  |  |  |
|  | Very Good | 81 | 22% | 79 | 22% | 81 | 22% |
|  | Good | 196 | 54% | 196 | 54% | 196 | 54% |
|  | Fair | 82 | 22% | 81 | 22% | 82 | 22% |
|  | Bad | 6 | 2% | 6 | 2% | 6 | 2% |
|  | Very Bad | 0 | 0% | 0 | 0% | 0 | 0% |
| Mental Health |  |  |  |  |  |  |  |
|  | Very Good | 73 | 20% | 72 | 20% | 73 | 20% |
|  | Good | 167 | 46% | 166 | 46% | 167 | 46% |
|  | Fair | 106 | 29% | 105 | 29% | 106 | 29% |
|  | Bad | 19 | 5% | 19 | 5% | 19 | 5% |
|  | Very Bad | 0 | 0% | 0 | 0% | 0 | 0% |

### Appendix D Table D.1: Sociodemographic characteristics of the sample by broadcaster

| *Variable* | *Detail* | *Total Sample* | | | *Watch ITV* | | | *Watch BBC* | |
| --- | --- | --- | --- | --- | --- | --- | --- | --- | --- |
|  |  | *Mean (SD)* | *Range* | *Mean (SD)* | | *Range* | *Mean (SD)* | | *Range* |
| Weekly Bets |  | 10 (14) | *[1, 150]* | 10 (14) | | *[1, 150]* | 10 (14) | | *[1, 150]* |
|  |  |  |  |  | |  |  | |  |
| Weekly Spending on Bets |  | £77.88 (£155.34) | [£1, £1500] | £78.22 (£155.88) | | [£1, £1500] | £77.88 (£155.34) | | [£1, £1500] |
|  |  |  |  |  | |  |  | |  |
| Number of Accounts |  | 6 (6.5) | *[1, 49]* | 6 (6.5) | | *[1, 49]* | 6 (6.5) | | *[1, 49]* |
|  |  |  |  |  | |  |  | |  |
|  |  | *Frequency* | *Percentage* | *Frequency* | | *Percentage* | *Frequency* | | *Percentage* |
|  |  |  |  |  | |  |  | |  |
| Gambling Risk Level |  |  |  |  | |  |  | |  |
|  | No risk | 95 | 26% | 95 | | 26% | 95 | | 26% |
|  | Lower Risk | 128 | 35% | 127 | | 35% | 128 | | 35% |
|  | Medium Risk | 103 | 28% | 101 | | 28% | 103 | | 28% |
|  | Higher Risk | 39 | 11% | 39 | | 11% | 39 | | 11% |
|  |  |  |  |  | |  |  | |  |
| Existing World Cup Bet |  |  |  |  | |  |  | |  |
|  | Yes | 217 | 59% | 215 | | 59% | 217 | | 59% |
|  | No | 148 | 41% | 147 | | 41% | 148 | | 41% |
| Betting Alone |  |  |  |  | |  |  | |  |
|  | Almost always | 120 | 33% | 119 | | 33% | 120 | | 33% |
|  | Most of the time | 157 | 43% | 156 | | 43% | 157 | | 43% |
|  | Sometimes | 85 | 23% | 84 | | 23% | 85 | | 23% |
|  | Never | 3 | 1% | 3 | | 1% | 3 | | 1% |
| Chosen Operator |  |  |  |  | |  |  | |  |
|  | Betfair | 41 | 11% | 40 | | 11% | 41 | | 11% |
|  | Sky Bet | 87 | 24% | 87 | | 24% | 87 | | 24% |
|  | Bet365 | 99 | 27% | 99 | | 27% | 99 | | 27% |
|  | Paddy Power | 31 | 8% | 31 | | 8% | 31 | | 8% |
|  | Ladbrokes | 24 | 7% | 24 | | 7% | 24 | | 7% |
|  | Coral | 16 | 4% | 16 | | 4% | 16 | | 4% |
|  | Betfred | 7 | 2% | 7 | | 2% | 7 | | 2% |
|  | LiveScore | 2 | 1% | 2 | | 1% | 2 | | 1% |
|  | William Hill | 49 | 13% | 48 | | 13% | 49 | | 13% |
|  | Other | 9 | 2% | 8 | | 2% | 9 | | 2% |
| Betting Types |  |  |  |  | |  |  | |  |
|  | Online betting on another sport/event | 359 | 98% | 357 | | 98% | 359 | | 98% |
|  | National Lottery | 241 | 66% | 239 | | 65% | 241 | | 66% |
|  | Online Games | 198 | 54% | 196 | | 54% | 198 | | 54% |
|  | Horse Races | 218 | 60% | 217 | | 59% | 218 | | 60% |
|  | Scratch Cards | 173 | 47% | 170 | | 47% | 173 | | 47% |
|  | Sports events (bookmakers) | 120 | 33% | 121 | | 33% | 120 | | 33% |
|  | Betting Exchange | 154 | 42% | 153 | | 42% | 154 | | 42% |
|  | Fruit/Slot Machines | 119 | 33% | 117 | | 32% | 119 | | 33% |
|  | Bingo | 72 | 20% | 71 | | 19% | 72 | | 20% |
|  | Football Pools | 51 | 14% | 50 | | 14% | 51 | | 14% |
|  | Virtual Gaming (bookmakers) | 54 | 15% | 53 | | 15% | 54 | | 15% |
|  | Dog Races | 38 | 10% | 38 | | 10% | 38 | | 10% |
|  | Table Games (Casino) | 70 | 19% | 69 | | 19% | 70 | | 19% |
|  | Poker in a tournament | 33 | 9% | 32 | | 9% | 33 | | 9% |
|  | Other events (bookmakers/phone) | 22 | 6% | 21 | | 6% | 22 | | 6% |
| Alcohol Risk Level |  |  |  |  | |  |  | |  |
|  | Low risk | 224 | 61% | 222 | | 61% | 224 | | 61% |
|  | Increasing risk | 109 | 30% | 109 | | 30% | 109 | | 30% |
|  | Higher risk | 30 | 8% | 29 | | 8% | 30 | | 8% |
|  | Possible dependence | 2 | 1% | 2 | | 1% | 2 | | 1% |

### Appendix D Figure D. 2: Gambling and other behavioural characteristics of the sample by broadcaster

**Note: Gambling risk level measured using the Problem Gambling Severity Index (PGSI): 0 “no risk” 1-2 “low-risk” 3-7 “medium-risk” 8+ “higher-risk” (or ‘problem’ gambler); “Alcohol risk level measured using the Alcohol Use Disorders Identification Test (AUDIT-C): 0-4 “low-risk” 5-7 “increasing-risk” 8-10 “higher-risk” 11-12 “possible dependence”. Participants could select multiple answers on the “betting types” question; *One participant responded that they had 0 betting accounts with different companies, which we have assumed means they only hold one account with one company.*

**Appendix E:**

**Betting and Advertising During Games**

##
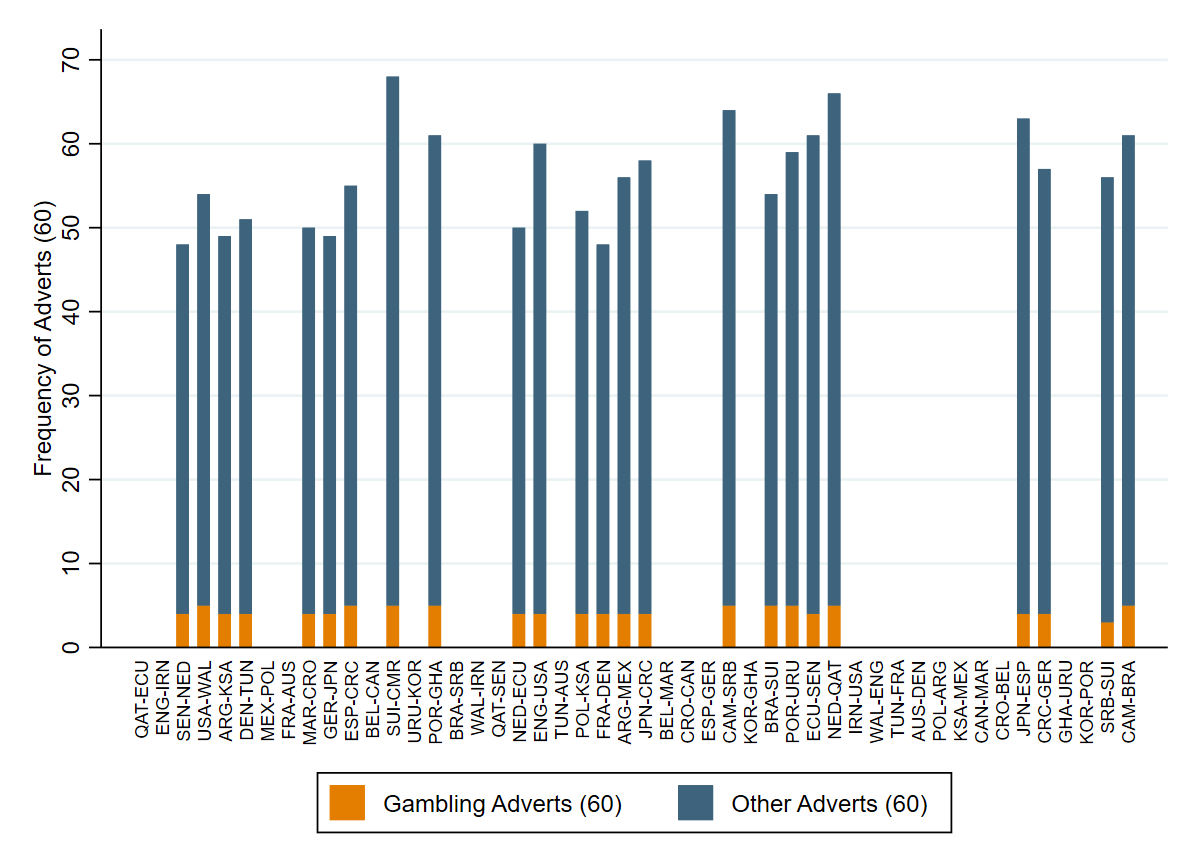

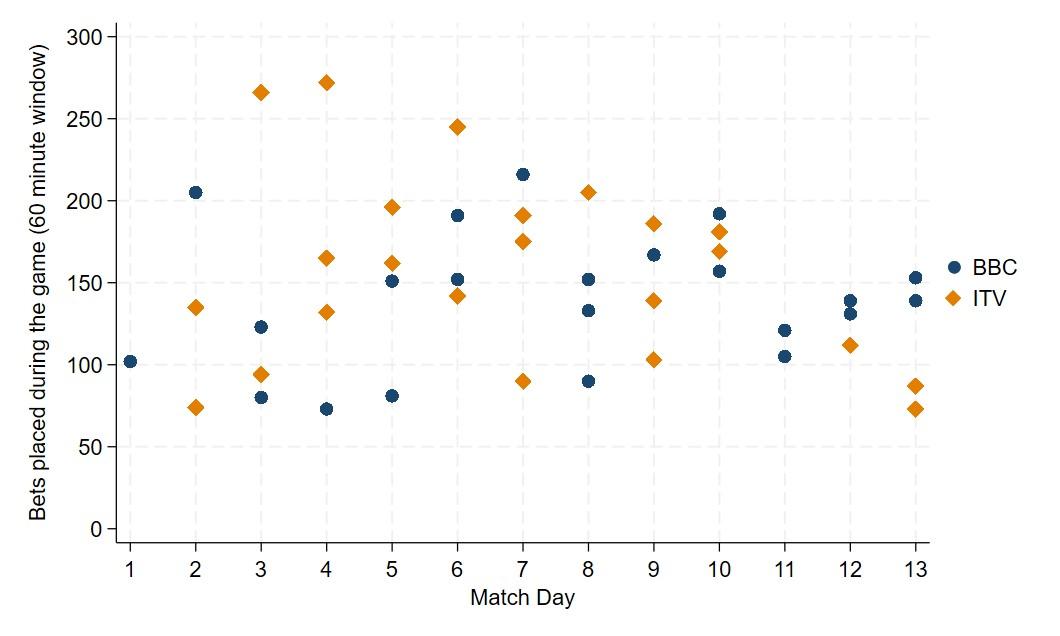
Appendix E: Betting and Advertising During Live Games

### Appendix E Figure E.2: The frequency of television advertisements by match (60-minute window)

### Appendix E Figure E.1: The frequency of bets placed during the game by match and broadcaster (60-minute window)

**Appendix F:**

**Robustness Checks**

## Appendix F: Robustness Checks

STATA does not conduct formal overdispersion tests for Poisson models with fixed effects. As a robustness check, Negative Binomial models were estimated, and model fit was compared using Akaike’s Information Criterion (AIC) and Bayesian Information Criterion (BIC). In the main models reported in this paper, robust standard errors are employed to account for any potential overdispersion.

|  | Negative Binomial 60 | Negative Binomial 30 | Negative Binomial 15 | Negative Binomial 10 |
| --- | --- | --- | --- | --- |
| **ITV** | **1.17^***^** | **1.19^***^** | **1.25^***^** | **1.27^***^** |
|  | **[1.11,1.24]** | **[1.12,1.28]** | **[1.16,1.35]** | **[1.17,1.38]** |
| Watch | 1.15^***^ | 1.15^***^ | 1.15^***^ | 1.16^***^ |
|  | [1.07,1.24] | [1.05,1.25] | [1.04,1.27] | [1.04,1.29] |
| Weekend | 0.98 | 0.96 | 0.96 | 0.96 |
|  | [0.91,1.05] | [0.88,1.05] | [0.87,1.06] | [0.87,1.07] |
| Evening | 0.57^***^ | 0.60^***^ | 0.50^***^ | 0.45^***^ |
|  | [0.53,0.61] | [0.55,0.65] | [0.45,0.56] | [0.40,0.50] |
| England | 1.45^***^ | 1.43^***^ | 1.31^***^ | 1.16 |
|  | [1.30,1.62] | [1.24,1.65] | [1.10,1.56] | [0.96,1.41] |
| Top Views | 0.80^***^ | 0.75^***^ | 0.78^***^ | 0.77^***^ |
|  | [0.75,0.85] | [0.70,0.82] | [0.71,0.85] | [0.70,0.85] |
| Match Length | 1.01^**^ | 1.01 | 1.00 | 0.99 |
|  | [1.00,1.02] | [1.00,1.02] | [0.98,1.01] | [0.98,1.01] |
| Bet on Match | 1.73^***^ | 1.62^***^ | 1.43^***^ | 1.34^***^ |
|  | [1.61,1.86] | [1.48,1.76] | [1.29,1.58] | [1.20,1.48] |
| Follow Match | 1.05^*^ | 1.02 | 1.04 | 1.03 |
|  | [1.00,1.09] | [0.97,1.08] | [0.98,1.11] | [0.96,1.10] |
| Diff in FIFA Ranking | 1.00 | 1.00 | 1.00^**^ | 1.00^**^ |
|  | [1.00,1.00] | [1.00,1.00] | [1.00,1.01] | [1.00,1.01] |
| Dispersion | 8.82^***^ | 7.30^***^ | 7.56^***^ | 8.20^***^ |
|  | [7.21,10.78] | [5.96,8.94] | [6.11,9.36] | [6.56,10.25] |
| Cluster Variability | 1.40^***^ | 1.36^***^ | 1.27^***^ | 1.21^**^ |
|  | [1.19,1.64] | [1.15,1.61] | [1.06,1.51] | [1.01,1.44] |
| Observations | 17520 | 17520 | 17520 | 17520 |

### Appendix F Table F. 1: Negative Binomial models

*Note: Key explanatory variable is a binary variable for the broadcaster (1 “ITV” 0 “BBC”); Negative Binomial “n” is the window around the game e.g. Negative Binomial 60 represents the 60-minute window; Coefficients are Incidence Rate Ratios (IRR) showing the change in the frequency of football bets placed ‘during the game’; These models employ random effects to allow for tests for overdispersion; Confidence intervals in parentheses; ^*^ p < 0.1, ^**^ p < 0.05, ^***^ p < 0.01.*

|  | **60** | | **30** | | **15** | | **10** | |
| --- | --- | --- | --- | --- | --- | --- | --- | --- |
|  | *AIC* | *BIC* | *AIC* | *BIC* | *AIC* | *BIC* | *AIC* | *BIC* |
| **Poisson** | 22092.99 | 22170.20 | 17356.24 | 17433.25 | 14216.60 | 14293.37 | 12991.10 | 13067.77 |
| **Negative Binomial** | 24486.19 | 24587.21 | 19357.49 | 19458.51 | 16125.12 | 16226.14 | 14908.07 | 15009.10 |

### Appendix F Table F.2: Akaike’s Information Criterion (AIC) and Bayesian Information Criterion (BIC) for Poisson and Negative Binomial models

The results indicate that, while there is evidence of some overdispersion and small between-cluster variability, the main findings are consistent across models. The Poisson fixed-effects models provide a better fit to the data, as reflected by lower AIC and BIC values. Minimal overdispersion, accounted for through fixed effects and robust standard errors supports the preference for the Poisson models.

Similarly, STATA does not compute variance inflation factors (VIFs) for fixed-effects Poisson models. Instead, pairwise correlations between the independent variables are presented to assess potential multicollinearity. There are no highly correlated variables which mitigate any multicollinearity concerns.

| Variables | ITV | Watch | Weekend | Evening | England | Top views | Match length | Match bet | Follow count | FIFA difference |
| --- | --- | --- | --- | --- | --- | --- | --- | --- | --- | --- |
| ITV | 1.000 |  |  |  |  |  |  |  |  |  |
| Watch | 0.010 | 1.000 |  |  |  |  |  |  |  |  |
| Weekend | 0.096 | 0.072 | 1.000 |  |  |  |  |  |  |  |
| Evening | 0.000 | 0.189 | 0.102 | 1.000 |  |  |  |  |  |  |
| England | -0.086 | 0.196 | 0.050 | 0.183 | 1.000 |  |  |  |  |  |
| Top views | 0.042 | 0.295 | 0.120 | 0.383 | 0.269 | 1.000 |  |  |  |  |
| Match length | 0.093 | -0.050 | -0.107 | -0.080 | -0.198 | -0.180 | 1.000 |  |  |  |
| Match bet | -0.012 | 0.376 | 0.053 | 0.184 | 0.240 | 0.243 | -0.060 | 1.000 |  |  |
| Follow count | 0.016 | 0.094 | 0.038 | 0.110 | 0.066 | 0.097 | 0.016 | 0.188 | 1.000 |  |
| FIFA difference | -0.014 | -0.096 | -0.322 | -0.117 | -0.217 | -0.159 | 0.344 | -0.077 | 0.021 | 1.000 |
|  | | | | | | | | | | |

### Appendix F Table F.3: Pairwise correlations between explanatory variable
